# Emerging status of anaplasmosis in cattle in Hisar

**DOI:** 10.14202/vetworld.2015.768-771

**Published:** 2015-06-21

**Authors:** Tarun Kumar, Neelesh Sindhu, Gaurav Charaya, Ankit Kumar, Parmod Kumar, Gauri Chandratere, Divya Agnihotri, Rajesh Khurana

**Affiliations:** 1 Teaching Veterinary Clinical Complex, Lala Lajpat Rai University of Veterinary & Animal Sciences, Hisar - 125 004, Haryana, India; 2 Department of Veterinary Medicine, Lala Lajpat Rai University of Veterinary & Animal Sciences, Hisar - 125 004, Haryana, India; 3 Department of Veterinary Pathology, Lala Lajpat Rai University of Veterinary & Animal Sciences, Hisar - 125 004, Haryana, India

**Keywords:** anaplasmosis, giemsa staining, hemoprotozoan, oxytetracycline

## Abstract

**Aim:**

*Anaplasma marginale* is a rickettsial pathogen responsible for progressive anemia in ruminants leading to huge economic losses. The objectives of this cross-sectional study were to determine the prevalence of anaplasmosis and therapeutic evaluation of traditional line of treatment.

**Materials and Methods:**

A total of 168 cattle presented to Teaching Veterinary Clinical Complex, Lala Lajpat Rai University of Veterinary & Animal Sciences, Hisar during the period of 3 months (July-September, 2014) with history of fever, anorexia, reduced milk yield and tick infestation were analyzed for prevalence of hemoprotozoan diseases using classical giemsa stained thin blood smear parasitological method.

**Results:**

Out of these 168 animals, 7 (4.17%) were found to be suffering from anaplasmosis on the basis of presence of dense, rounded, intra-erythrocytic bodies situated on or near the margin of the erythrocytes. Overall prevalence of theileriosis and babesiosis were found to be 42.9% and 1.8%, respectively. Level of parasitemia was noticed to be 1.2%, 0.8% and 0.9% in babesiosis, theileriosis, and anaplasmosis, respectively. The most marked and common clinical signs reported in all the cases were severe anemia (hemoglobin=3-6 g/dl) and history of fever, followed by normal body temperature. Following treatment with oxytetracycline parenterally along with supportive therapy out of seven cases six got recovered without any side-effects.

**Conclusion:**

The current study indicates the emerging status of anaplasmosis in this part of the country as during the past few years there are very few reports showing the prevalence of clinical cases of anaplasmosis. Treatment with oxytetracycline yielded excellent result showing recovery in most of the clinical cases.

## Introduction

Anaplasmosis, formerly known as gall sickness is a tick-borne disease caused by an obligate intraerythrocytic rickettsial microorganism, *Anaplasma marginale* and *Anaplasma centrale* of the order *Rickettsiales*, family *Anaplasmataceae*. Cattle, sheep, goats, buffalo, and some wild ruminants can be infected with the erythrocytic *Anaplasma* but cattle have been found to be more susceptible to *Anaplasma* infection than the buffaloes [1]. *Anaplasma* is transmitted by at least 20 ticks of various species [2,3], including *Hyalomma* spp., *Rhipicephalus* spp., *Boophilus* spp., *Ixodes* spp., *Demacentor* spp., [4] however *Boophilus microplus* is found to be the major transmitting agent [5]. Mechanical transmission by biting flies or blood-contaminated fomites act as alternative means of spread.

Disease is mainly characterized by progressive hemolytic anemia associated with fever, jaundice, decreased milk production, abortions, hyperexcitability and in some cases sudden death [6,7]. Despite recent advances for diagnosis of bovine anaplasmosis and other hemoprotozoan from clinical samples, classical giemsa stained thin blood smear (GSTBS) parasitological method is a gold standard test for early, easy and economic detection of parasite. For therapeutic management, administration of tetracycline antibiotics along with supportive medication including vitamin C, parenteral hematinics and liver extract ensures complete and smooth recovery.

In continuation with the limited existing knowledge, present study was planned to estimate (a) Prevalence of anaplasmosis in clinical cases suspected for hemoportozoan infection and (b) To determine the efficacy of oxytetracycline in the treatment of anaplasmosis in cattle.

## Materials and Methods

### Ethical approval

The blood samples used in the current study were taken from clinical cases brought to Teaching Veterinary Clinical Complex (TVCC). As per University rules, for these samples approval of Institutional Animal Ethics Committee is not required.

### Samples

A total of 168 blood samples from cattle were collected in the months of July 2014 to September 2014 primarily having a history of tick infestation, fever, jaundice and pale mucous membrane. Blood samples were collected from a jugular vein in ethylenediaminetetraacetic acid containing vials for complete blood count and Giemsa staining. A questionnaire based on information regarding breed, gender, age, presence of ticks, previous treatment and herd size was collected to exemplify their correlation with the study.

### Area descriptions

Present work was carried out in Teaching Veterinary Clinical Complex, College of Veterinary Sciences, Lala Lajpat Rai University of Veterinary and Animal Sciences, Hisar.

### Giemsa staining

Thin blood smears were prepared immediately after blood collection. Blood smears were labeled, air-dried, fixed with methanol, stained with giemsa stain and examined microscopically for presence of *A. marginale, Theileria* spp. and *Babesia* spp. in erythrocytes. Examination of the smears was performed at 100× magnification with compound microscope by searching at least 50 fields per slide. The parasites were identified as described by OIE [8].

### Treatment

Animals found positive for anaplasmosis were treated with oxytetracycline intravenously (10 mg/kg) daily for 3 days along with vitamin C, parenteral hematinics and liver extract. Response to treatment was assessed 7^th^ day post-treatment by repeated blood sampling.

## Results

### Clinical findings

All animals were acutely infected and were having peculiar clinical signs like enlarged lymph nodes, pale mucous membrane, inappetence, weakness, increased rectal temperature and respiratory distress as described in Table-1.

**Table-1:** Clinical signs and laboratory findings of cattle affected with anaplasmosis.

Age	Rectal temperature (°F)	Hb (g%)	TLC/µl	DLC				

				N	L	B	E	M
3 years33	102.5	4	4400	40	60	-	-	-
25 days	105	3.8	2700	70	30	-	-	-
6 years	102.6	6	5500	65	35	-	-	-
7 years	102.8	3	5800	62	32	-	6	-
6 years	103.8	4.5	6200	60	40	-	-	-
30 days	104	4.0	4800	72	28	-	-	-
4 years	103	3.6	9600	43	51	-	6	-

*Hb=Hemoglobin, TLC=Total leukocyte count, N=Neutrophils, L=lymphocytes, B=Basophils, E=Eosinophils, M=Monocytes

### Parasitic load

**Figure-1:**
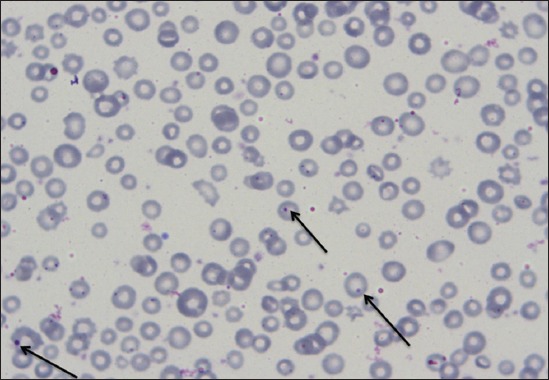
Dense, rounded, intra erythrocytic bodies situated on or near the margin of the erythrocytes.

**Figure-2:**
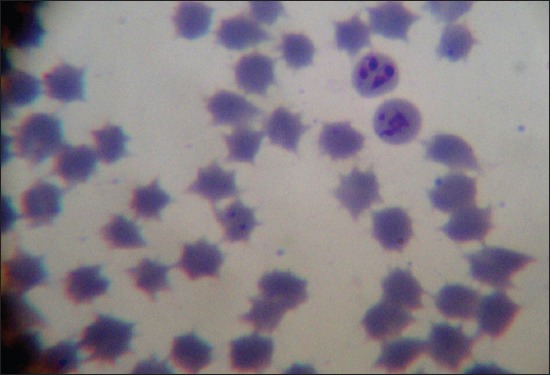
Giemsa-stained *Babesia bigemina*-infected erythrocytes.

**Figure-3:**
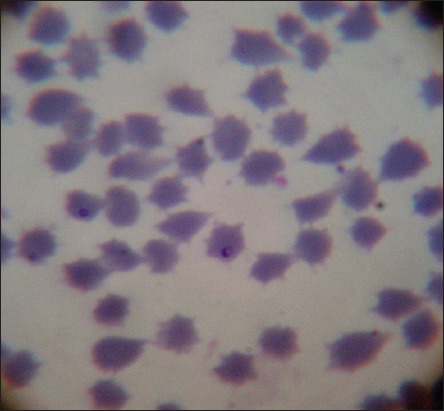
Microscopic examination of giemsa stained blood smear showing piroplasm of theileria parasite in erythrocyte.

Out of 168 animals, 82 (48.80%) were found positive for hemoparasite infection based on giemsa staining technique. Thorough examination of slide stained with giemsa revealed zero mixed infection found in the present study. Increased percentage of hemoparasite infection can be correlated with prevalence of more number of vector populations in the rainy season (July-August). Among 82 positive samples 72 (87.80%), 7 (8.54%) and 3 (3.66%) were positive for theileriosis, anaplasmosis, and babesiosis, respectively (Table-2). Level of parasitemia was noticed to be 1.2%, 0.8% and 0.9% in babesiosis, theileriosis, and anaplasmosis, respectively. Overall prevalence of anaplasmosis was found to be 4.17%. *Anaplasma* spp. in the blood smear was observed as dense, rounded, intra-erythrocytic bodies situated on or near the margin of the erythrocytes (Figure-1). Babesia species were identified as a pair of piroplasm covering more than half of erythrocyte (Figure-2). Theileria species were identified as signet ring shaped or comma-shaped piroplasm inside the erythrocytes (Figure-3).

**Table-2:** Prevalence of hemoprotozoan infections in cattle in Haryana.

Month	Tested	Positive			Total positive

		*Theileria* spp.	*Babesia* spp.	*Anaplasma* spp.	
July	62	32	1	1	34
August	63	22	2	5	29
September	43	18	0	1	19
Total	168	72	3	7	82
Percent positive	100	42.85	1.78	4.17	48.80

Prevalence of hemoprotozoan infections in different months is shown in Table-2, indicating an abrupt rise in the prevalence of *Anaplasma* spp. in the month of august. Cattle affected with anaplasmosis were cross bred. Out of seven animals positive for anaplasmosis, three (42.8%) were calves in the age group from 25 days to 40 days.

### Hematological analysis

All seven cases infected with anaplasmosis showed hematological values on lower side. Hemoglobin estimation revealed severe anemia in all infected cases with hemoglobin ranging from 3.0 g% to maximum of 6%, whereas total leukocyte count ranges from 4400 to 9600 per µL (Table-1).

### Treatment response

Clinical picture after 1 week of treatment showed improvement in appetite, the mucous membrane was light pink in color and body temperature was normal. Post-treatment sampling after 1 week of all the treated animals showed increase in blood parameters (hemoglobin and total leukocyte count).

### Side effect

No severe side effect was observed following treatment.

## Discussion

Bovine anaplasmosis, a rickettsial disease caused by *A. marginale* is responsible for great economic losses in developing countries like India, where it is highly endemic [9]. Anaplasmosis has been reported in the cattle population of all age groups around all six populated continents in world [10]. Higher prevalence of disease in calves in present study might be due to underdeveloped immune system at young age, less or absence of exposure to parasitic infections, softer skin resulting in an ease of mouth parts penetration of vector or lower disease resistance in young ones as compared to adults. However Khan *et al*. [11] and Atif *et al*. [12] found adults to be more affected than calves. In the present study, the diagnosis of anaplasmosis in cattle was based on case history (tentatively) and microscopic examination for confirmation in clinical cases using GSTBS parasitological method. GSTBS parasitological method gives a clear picture about the parasite and cannot give a false positive results. However, recent molecular technology have gained a lot of importance in the diagnosis of hemoparasitic infection, but they being costlier, time-consuming and requires expertise limit their use. High prevalence of disease in hot and humid climatic conditions in present study might be attributed to the restricted movement of animals due to confinement as well as because of tick breeding season resulting in abrupt increase in anaplasma infection. All the cases presented were acutely affected and animals were brought to Teaching Veterinary Clinical Complex, Hisar. Comparing the overall prevalence of anaplasmosis, it was found in close agreement with Sajid *et al*. [13], whereas Ashuma *et al*. [14] found prevalence of anaplasmosis to be on higher side (11.25%) as compared to the present study (4.17%). As there was no previous report of anaplasmosis from TVCC, Hisar, Haryana, in the recent past, so the disease can be categorized under the emerging status. Disease prevalence increased might be due to precipitation of various factor and we hereby reporting the disease prevalence indicating a threat to the dairy industry. Level of parasitemia (5.9%) shown by Ashuma *et al*. [14] was found to be on higher side as compared to parasitemia in the present study (0.9%). There was no characteristic difference in the level of parasitemia and clinical manifestation. Kumar and Sangwan [15] too showed high prevalence of *A. marginale* (46.9%) under different cattle management systems in Haryana but described absence of clinical cases in cattle highlighting the endemic stability of anaplasmosis in the state. Classical treatment of anaplasmosis as described in the present study proved to be effective.

## Conclusion

Increased clinical cases of anaplasmosis during the rainy season of the year 2014 showed an emerging status of this economically important disease. Hemoglobin was significantly low in all the affected cases, and oxytetracycline again proved to be a most effective drug in the present scenario.

## Authors’ Contributions

TK and NS proposed the study. GC, AK and PK carried out the blood collection and hematological examination part. AK and PK did the therapeutic management. GC and DA critically observed the slide and identification of slide. GC, TK and NS prepared the manuscript. Finalization of manuscript was done by RK. All authors read and approved the final manuscript.
